# Varicella-zoster virus ORF 58 gene is dispensable for viral replication in cell culture

**DOI:** 10.1186/1743-422X-5-54

**Published:** 2008-04-30

**Authors:** Hironori Yoshii, Kay Sadaoka, Masaaki Matsuura, Kazuhiro Nagaike, Michiaki Takahashi, Koichi Yamanishi, Yasuko Mori

**Affiliations:** 1 Laboratory of Virology and Vaccinology, Division of Biomedical Research, National Institute of Biomedical Innovation, Osaka, Japan; 2 Kanonji Institute, the Research Foundation for Microbial Diseases of Osaka University, Kanonji, Kagawa, Japan; 3 The Research Foundation for Microbial Diseases of Osaka University, Suita, Osaka, Japan

## Abstract

**Background:**

Open reading frame 58 (ORF58) of varicella-zoster virus (VZV) lies at the 3'end of the Unique long (U_L_) region and its functional is unknown. In order to clarify whether ORF58 is essential for the growth of VZV, we constructed a deletion mutant of ORF58 (pOka-BACΔ58) from the Oka parental genome cloned into a bacterial artificial chromosome (pOka-BAC).

**Results:**

The ORF58-deleted virus (rpOkaΔ58) was reconstituted from the pOka-BACΔ58 genome in MRC-5 cells, indicating that the ORF58 gene is non-essential for virus growth. Comparison of the growth rate of rpOkaΔ58 and recombinant wild-type virus by assessing plaque sizes revealed no significant differences between them both in MRC-5 cells and malignant melanoma cells.

**Conclusion:**

This study shows that the ORF58 gene is dispensable for viral replication and does not affect the virus' ability to form plaques *in vitro*.

## Background

Varicella-zoster virus (VZV) is a member of the herpesviridae family, and its primary infection causes varicella in children. VZV often persistently infects dorsal root ganglia (DRG) and is sometimes activated from a latent to lytic state, causing zoster in aged and immunosuppressed individuals [1]. The double-stranded VZV genome contains approximately 125 kbp with at least 71 open reading flames (ORFs) [2]. Although understanding VZV virulence and attenuation mechanisms requires study of the VZV-encoded genes, little has been reported on VZV genes compared with those of herpes simplex virus (HSV).

The ORF58 of VZV lies at the 3'end of the Unique long (U_L_) region and its function is unknown. Although ORF57, its neighboring gene, is dispensable in cell culture [3], there has been no report yet on ORF58. Therefore, to investigate the functional roles of this gene in VZV infection, we constructed an ORF58-deletion mutant of VZV, and analyzed its susceptibility in both MRC-5 cells and malignant melanoma cells.

## Results and Discussion

**Figure 1:**
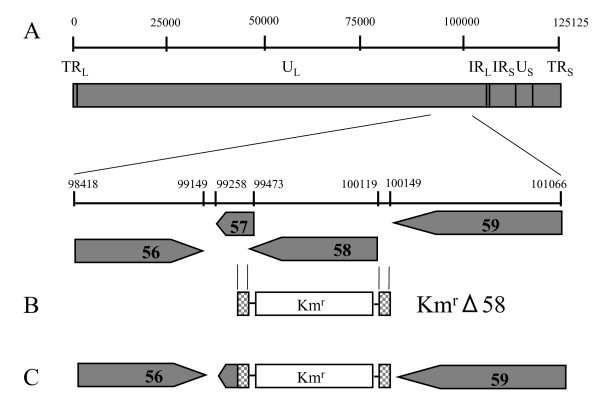
**Construction of recombinant virus rpOkaΔ58**. (A) The VZV genome consists of two unique regions (U_L _and U_S_) and of inverted repeat sequences (IR_L_, IR_S_, and TR_Ss_) flanking the U_S _region. An enlarged section shows the analyzed portion of the genome, containing open reading frame (ORF) 56, 57, 58 and 59. ORFs are drawn as pointed rectangles. (B) A fragment consisting of the 3'UTR of ORF58, the kanamycin-resistance gene (km^r^), and the 5'UTR of ORF58 was amplified by PCR and used for mutagenesis of an infectious full-length pOka genome in *E. coli *and named Km^r^Δ58. (C) The entire ORF58 gene was replaced by the kanamycin-resistance gene in *E. coli*. TR_L_, terminal repeat long; U_L_, unique long; IR_L_, internal repeat long; IR_S_, internal repeat short; U_s_, unique short; TR_S_, terminal repeat long.

We produced the deletion mutant of the ORF58 gene by using the BAC system [4]. The deletion mutant of the ORF58 gene (pOka-BACΔ58) was constructed by recombination in *E. coli *harboring pOka-BAC DNA [5] and pGETrec [6] with a fragment containing the kanamycin-resistance gene flanked by the 3'-UTR and 5'-UTR of the ORF58 gene. The pGETrec was kindly provided by Dr. Panayiotis A Ioannou. Thus, the ORF58 gene in the pOKa-BAC genome was replaced by the kanamycin-resistance gene (Fig. 1A, B, and 1C).

**Figure 2:**
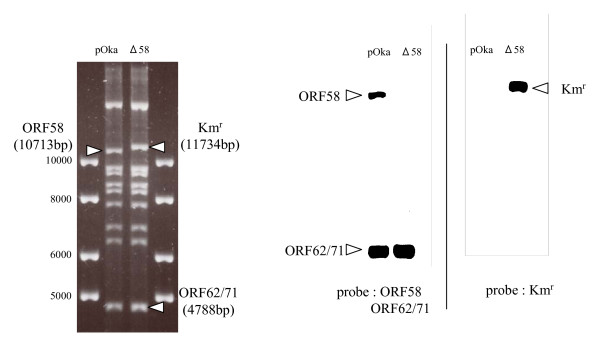
**Southern blotting analysis of pOka-BAC and pOka-BACΔ58. DNA**. Purified pOka-BAC DNA and pOka-BACΔ58 DNA were digested by *Bam*HI and loaded onto a 0.5% TBE agarose gel. The fragments recognized by the ORF58, ORF62/71 and Km^r ^probes (right) are indicated by arrowheads in the photograph (left). Southern blotting was performed using ORF58, ORF62/71, or the Km^r ^gene as a probe.

The recombination was confirmed by Southern blotting using a fragment of the internal sequence of the ORF58 gene, the ORF62/71 gene, or the kanamycin-resistance gene as a probe (Fig. 2). As shown in Figure 2, the signal for the ORF 58 gene was detected in the pOka-BAC genome but not in the pOka-BACΔ58 genome. The signal for the ORF62/71 gene, used as a positive control, was detected in both genomes, and the signal for the kanamycin-resistance gene was detected in the pOka-BACΔ58 genome but not in the pOka-BAC genome. The recombination was also confirmed by PCR using primer pairs that annealed to the internal region of the kanamycin-resistance gene and the external region of ORF58 (data not shown). The results confirmed that the ORF58 gene was properly replaced by the kanamycin-resistant gene in the pOka genome.

**Figure 3:**
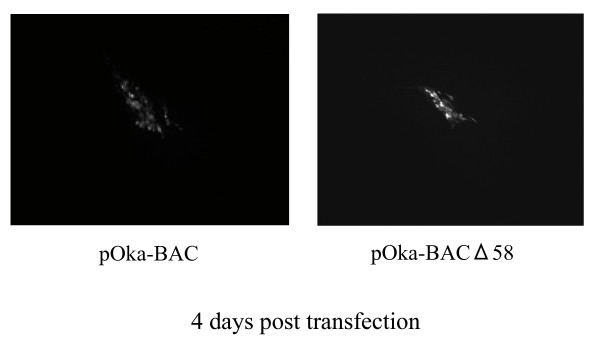
**Reconstitution of the infectious recombinant virus**. (A) MRC-5 cells were transfected with purified pOka-BAC DNA or pOka-BACΔ58 DNA. Four days after transfection, typical CPEs, which fluoresce under the exciting light, were observed in the cells transfected with either pOka-BAC DNA or pOka-BACΔ58 DNA.

We next examined whether the ORF58 gene was essential for the replication of VZV in MRC-5 cells. To reconstitute the virus from its genome, MRC-5 cells were transfected with pOka-BAC or pOka-BACΔ58 DNA (Fig. 3). At 4 days post-transfection, typical cytopathic effects (CPEs), which fluoresce under the exciting light, was observed in the MRC-5 cells transfected with the pOka-BACΔ58 DNA, as well as with the pOka-BAC DNA (Fig. 3), suggesting that the ORF58 gene is dispensable for viral replication in cell culture.

Before performing the remaining experiments, in order to exclude the possibility to affect the packaging of viral genome, we deleted the BAC sequences from the recombinant viruses derived from pOka-BAC and pOka-BACΔ58 using the Cre-loxP system [7] (data not shown), and the resulting viruses were named rpOka and rpOkaΔ58, respectively.

**Figure 4:**
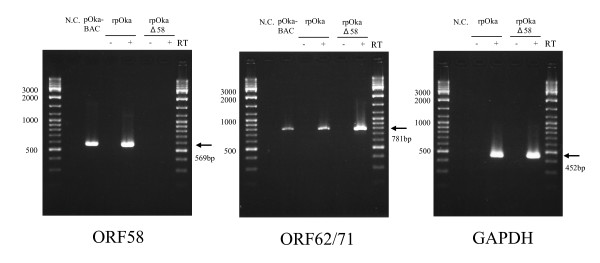
**Confirming the expression of ORF58 by RT-PCR**. rpOka or rpOkaΔ58-infected cells were harvested at 24 hrs p.i., and the RNAs were extracted. The cDNAs were synthesized from each RNA using Superscript III (Invitrogen), and PCRs were performed using the cDNAs as templates. PCRs were also performed with RT(-) to avoid amplification of contaminating genomic DNA. The size of each product is indicated by an arrow at the right of each panel. The molecular size (bp) is shown on the left of each panel. N.C.: negative control.

Next, to confirm that rpOkaΔ58-infected cells did not express the ORF58 gene, we performed RT-PCR using the cDNA from rpOka- and rpOkaΔ58-infected MRC-5 cells as a template. As shown in Figure 4, ORF58 cDNA was amplified from the rpOka-infected cells, but not from the pOkaΔ58-infected cells, indicating that the ORF58 gene product was not expressed in the rpOkaΔ58-infected cells. As positive controls, the ORF62/71 and GAPDH cDNAs were both amplified in both types of infected cells (Fig. 4).

**Figure 5:**
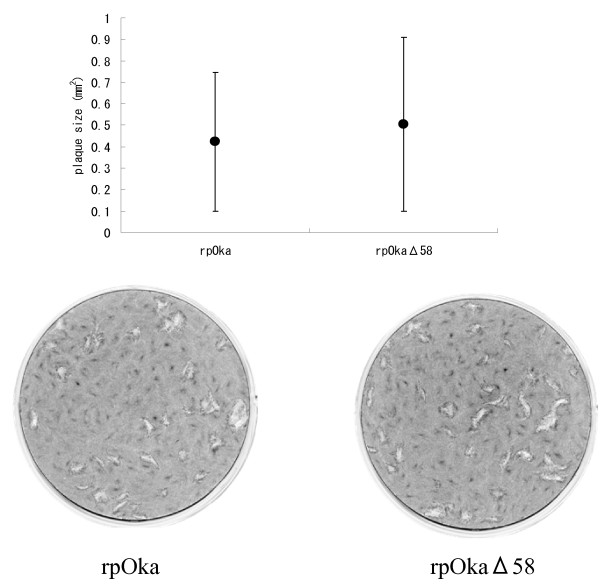
**Plaque size comparison between rpOka and rpOkaΔ58 in MRC-5 cells**. Plaque sizes after infection with cell-free rpOka and rpOkaΔ58 are indicated graphically (upper) and in photographs (lower). MRC-5 cells were infected with each cell-free virus and cultured for 10 days. The cells were fixed and stained with 1% crystal violet/70% EtOH and the sizes of 38 plaques (rpOka) and 41 plaques (rpOkaΔ58) were calculated and analyzed using ImageJ software (NIH, USA). Error bars in the graph indicate the standard deviation (SD).

Since the deletion mutant rpOkaΔ58 was able to be reconstituted, we next analyzed its ability to form plaques of rpOkaΔ58 with that of rpOka. Cell-free rpOka or rpOkaΔ58 virus was used to infect MRC-5 cells at approximately 50 PFU/well, and the resulting plaque sizes were compared (Fig. 5) at 10 days post infection (pi), after the infected cells were fixed and stained. As shown in Figure 5, no significant difference was observed between the plaque sizes resulting from infection with the two viruses (p > 0.05, Student's *t*-test).

**Figure 6:**
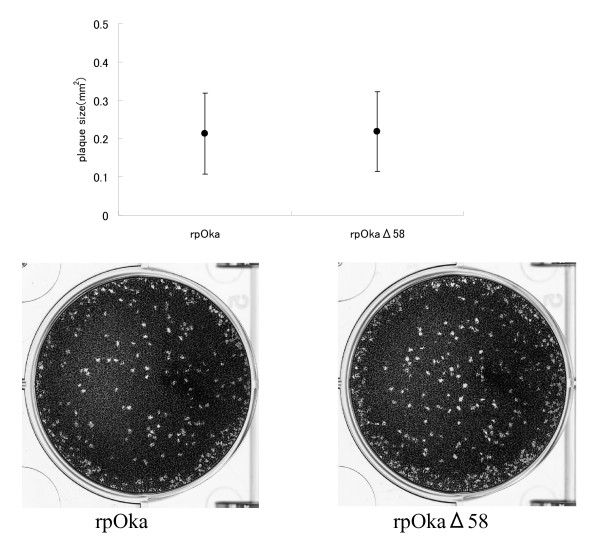
**Plaque size comparison between rpOka and rpOkaΔ58 in MeWo cells**. Plaque sizes after infection with cell-free rpOka and rpOkaΔ58 are indicated graphically (upper) and in photographs (lower). MeWo cells were infected with each cell-free virus and cultured for 10 days. The cells were fixed and stained with 1% crystal violet/70% EtOH and the sizes of 282 plaques (rpOka) and 208 plaques (rpOkaΔ58) were calculated and analyzed using ImageJ software (NIH, USA). Error bars in the graph indicate the standard deviation (SD).

In order to confirm whether rpOkaΔ58 grow in another cell lines, human malignant melanoma cell line, MeWo cells, were infected with these viruses. As shown in Figure 6, no significant difference of their plaque sizes was observed between the two viruses (p > 0.05, Student's *t*-test), suggesting that the ORF58 gene of VZV genome is dispensable for viral replication and does not affect virus growth in both cells. These results suggesting that the ORF58 gene of VZV genome is dispensable for viral replication and does not affect virus growth *in vitro*.

We have succeeded in deleting the entire ORF58 gene from the VZV genome using the BAC system. Infectious viruses could be reconstituted from the ORF58 deletion mutant, and the reconstituted viruses had similar growth kinetics to wild-type VZV in cell culture.

In this study, the N terminus of ORF57 was deleted in the process of deleting ORF58, because the C terminus of ORF58 overlaps with the N terminus of ORF57. The ORF57 gene product has been shown to be expressed in the cytosol and to be dispensable for viral growth in cell culture [3]. Therefore, we were not concerned that any observed effects would reflect the loss of the ORF57 N-terminus.

In investigations of VZV ORFs, the SCID-hu system has been used to assess the *in vivo *growth and tropism of VZV mutants constructed in cosmid systems [8-17] and BAC [18].

In HSV-1 and HSV-2, UL3 is the positional homologue of ORF58. The UL3 gene product is a phosphoprotein that is localized to the cytoplasmic and nuclear portions of HSV-1-infected cells [19]. In HSV-2-infected cells, the UL3 gene product localizes to the nucleus at the early stage of infection [20]. Whether the ORF58 gene product possesses similar characteristics to UL3 remains unknown. Further study will be required to demonstrate the localization and possible role of the ORF58 gene product in VZV infection.

## Conclusion

Here we show that the ORF58 gene is dispensable for viral replication and that the deletion mutant, rpOkaΔ58, grows as same as wild-type VZV in both MRC-5 cells and malignant melanoma cells. Construction and investigation of deletion mutants utilizing BAC system will make it easier to understand the virulence and attenuation mechanisms of VZV.

## Methods

### Cells and viruses

MRC-5 cells were cultured with modified minimum essential medium (MEM) supplemented with 10% fetal bovine serum (FBS). MeWo cells were cultured with Dulbecco's modified eagle medium (DMEM) supplemented with 10% FBS. pOka possessing BAC sequence were obtained previously[5]. Recombinant VZV was propagated by inoculation of MRC-5 cells with virus-infected cells.

### Generation of virus deletion mutants

VZV ORF58 was deleted within *Escherichia coli (E. coli) *by homologous recombination using ET recombinase. pOka-BAC clone which containing pOka full genome was generating as described previously [5].

*E. coli *harboring pOka-BAC DNA was transformed by pGETrec plasmid which express ET recombinase(kindly provided by Dr. Panayiotis A Ioannou, Murdoch Children's Research Institute, Department of Pediatrics, The University of Melbourne, Royal Children's Hospital) using MicroPulser electroporator(BIO-RAD).

To introduce homologous recombination, PCR reaction was performed in order to generate [flanking-kanamycin^R^-flanking] fragment using pCRII-TOPO plasmid (Invitrogen) as template. Primer pairs were designed as follows ; Forward primer(ACAAATTTCTGATGTTCCCCCGGCGTGGCAACGCTGGCATTTCCAAACACAGAAGTTCCTATTCTCTAGAAAGTATAGGAACTTCAGCAAGCGAACCGGA  ATTGC) contains homologous sequence of the upstream of ORF58(ACAAATTTCTGATGTTCCCCCGGCGTGGCAACGCTGGCATTTCCAAACACA) as flanking sequence, FRT sequence(GAAGTTCCTATTCTCTAGAAAGTATAGGAACTTC, single under line), homologous sequence of kanamycin resistant gene within pCRII-TOPO plasmid(AGCAAGCGAACCGGAATTGC, double under line). Reverse primer (GATCGATTGGAGTGTTATATAACACTCCAATCGACCCTCTCGCGTACCATGAAGTTCCTATACTTTCTAGAGAATAGGAACTTCCTTTTTCAATTCAGAAGAACTC) contains homologous sequence of the downstream of ORF58 (GATCGATTGGAGTGTTATATAACACTCCAATCGACCCTCTCGCGTACCAT) as flanking sequence, FRT sequence (GAAGTTCCTATACTTTCTAGAGAATAGGAACTTC, single under line), homologous sequence of kanamycin resistant gene within pCRII-TOPO plasmid (CTTTTTCAATTCAGAAGAACTC, double under line).

PCR products were transformed into E. coli harboring pOka-BAC DNA and pGETrec plasmid. The recombined clones were selected by chloramphenicol/kanamycin on LB plates and the correct recombination was confirmed by PCR (data not shown).

### Preparation of pOka-BAC and pOka-BACΔ58 genome

pOka-BAC and pOka-BACΔ58 genome was isolated using a NucleoBond BAC 100 kit (Macherey-Nagel) following the manufacturer's protocol.

### Reconstitution of infectious virus

One μg of pOka-BAC or pOka-BACΔ58 genome was transfected into MRC-5 cells by electroporation using a Nucleofection unit (Amaxa biosystems). The cells were then cultured with MEM supplement with 10% FBS for 4 days. To remove BAC sequence, MRC-5 cells were first infected with a recombinant adenovirus, AxCANCre, which expresses the Cre recombinase (kindly provided by Dr. Yasushi Kawaguchi, University of Tokyo). Twenty-four hrs later, the cells were super-infected with the recombinant viruses obtained from pOka-BAC genome(rpOka-BAC) or pOka-BACΔ58 genome(rpOka-BACΔ58), and cultured until plaques without GFP appeared. The plaques without GFP were isolated using glass isolation cups and transferred onto newly prepared MRC-5 cells to obtain BAC-deleted viruses, rpOka or rpOkaΔ58. After several rounds of isolation, cell-free viruses were obtained by sonicating the rpOka or rpOkaΔ58-infected cells and stored at -80°C.

### Southern blotting

Genome DNA of pOka-BAC and rpOkaΔ58 were extracted from *E. coli*. One μg of both DNAs were digested with *Bam*HI and loaded onto 0.5% agarose gel and electrophoresis was performed in 0.5 × TBE (44.5 mM Tris, 44.5 mM Borate, 1 mM EDTA). At 72 hour later, DNA fragments were transferred to Hybond N^+ ^nylon membrane(GE healthcare) with 0.4 N NaOH followed by washing with 2 × SSC (300 mM NaCl, 30 mM Na_3_-citrate). Hybridization and detection were performed using ECL direct labeling and detection system (GE healthcare). Probes used to detect ORF58, ORF62 and kanamycin resistant gene were labeled using the system following manufacture's protocol. The primer pairs used to create probes were: ORF58, VZ58F (aggacacgatctaaagccgt) and VZ58R (tccgtaccgacggcattgct); ORF62/71, G62N4 (gatcaaagcttagcgcag) and G62R4 (cctatagcatggctccag); kanamycin-resistance gene, KMF (atgattgaacaagatggattg) and KMR (aagaaggcgatagaaggcgatg). The transferred membrane was treated with hybridization buffer for 2 hours at 42°C followed by hybridization with the labeled probes for 18 hours at 42°C following manufacture's protocol, then was washed with primary wash buffer (6 M urea, 0.4% SDS and 0.5 × SSC) for 4 times at 42°C followed by washing with secondary wash buffer (2 × SSC), and the signals were detected with ECL detection reagents (GE healthcare) followed by exposing to X-ray film.

### RT-PCR

rpOka or rpOkaΔ58-infected cells were harvested at 24 hours p.i. Cells were extracted with 1 mL of TRIzol Reagent (Invitrogen) and 200 μL of chloroform. Cell extract was centrifuge and supernatant was added with 500 μL of isopropanol. Nucleic acid containing total RNA was obtained by centrifuge and resolved with 20 μL of DEPC-treated water. Seven μL of solution was added with 2 μL of 10× DNase buffer and 1 μL of DNaseI and incubated for 20 minutes followed by added with 1 μL of 25 mM EDTA and incubated at 60°C for 20 minutes thereafter transferred on ice. Eight micro litters of solution was added with 1 μL of oligo(dT)_15 _and 4 μL of dNTP(2.5 mM each) and incubated at 65°C for 5 minutes thereafter incubated on ice for 5 minutes. Solution was then added with 4 μL of 5× buffer, 1 μL of 0.1 M DTT, 1 μL of RNase inhibitor and 1 μL of SuperScriptIII reverse transcriptase(Invitrogen) and incubated at 50°C for 60 minutes followed by incubated at 70°C for 15 minutes. Single stranded RNA was digested from DNA/RNA hybrid by adding 0.5 μL of RNaseH and incubated at 37°C for 20 minutes.

Expression of mRNAs were confirmed using primers set anneal to ORF58 (forward primer: VZ58F (aggacacgatctaaagccgt), reverse primer: VZ58R (tccgtaccgacggcattgct)), ORF62 (forward primer: G62N4 (gatcaaagcttagcgcag), reverse primer: G62R4 (cctatagcatggctccag)) and GAPDH (forward primer: G3PDHF (accacagtccatgccatcac), reverse primer: G3PDHR (tccaccaccctgttgctgta)). pOka-BAC DNA was used as positive control.

### Comparison of Plaque sizes

VZV recombinants were assessed for the property of cell-to-cell spread by comparison of plaque sizes. Briefly, MRC-5 cells or MeWo cells were infected with approximately 50 PFU of cell-free viruses of rpOka or rpOkaΔ58, which was produced from pOka-BAC or pOka-BACΔ58 genome. The cells were cultured for 7 days at 37°C followed by stained with 1% crystal violet/70% ethanol. Plaque sizes were calculated with ImageJ software (NIH, USA).

## Competing interests

The authors declare that they have no competing interests.

## Authors' contributions

HY and YM designated research; HY, KS, MM and KN performed research; HY, MT, KY and YM analyzed data; and HY and YM wrote the paper.
